# 2734. Evaluation of Empiric Antimicrobial Therapy for End-Stage Liver Disease Patients at an Academic Medical Center

**DOI:** 10.1093/ofid/ofad500.2345

**Published:** 2023-11-27

**Authors:** Annie Kim, Emily A Kaip, David Quan, Sarah B Doernberg, Ripal Jariwala

**Affiliations:** Zuckerberg San Francisco General Hospital and Trauma Center, San Francisco, California; University of California, San Francisco Medical Center, San Francisco, California; UCSF Medical Center, San Francisco, California; University of California, San Francisco, San Francisco, California; UCSF Medical Center, San Francisco, California

## Abstract

**Background:**

Patients with end-stage liver disease (ESLD) are prone to decompensation secondary to infections, with an increasing prevalence of multidrug-resistant organisms (MDROs). These patients often receive broad-spectrum antimicrobials prior to transplant, pre-disposing them to resistant pathogens. Our study assessed institutional guideline adherence, antimicrobial prescribing, and the incidence of MDROs in this population.

**Methods:**

This IRB-approved, retrospective chart review included pre-transplant inpatients with ESLD on the adult liver transplant service who received antimicrobials between 1/1/20 and 10/30/20. Patients were excluded if they started antimicrobials post-transplant or transferred from another institution on parenteral antimicrobials. Per institutional guidelines for empiric antimicrobials in suspected infection in ESLD, patients were categorized into three infectious risk categories: Floor/Non-High-Risk (F-NHR), Floor/High-Risk (F-HR), and ICU or MELD greater than 35 (ICU/M35). Antimicrobial prescribing in each group was evaluated for guideline adherence and microbiology data. Results were analyzed using descriptive statistics.

**Results:**

We included 92 patients: 28 F-NHR, 50 F-HR, and 14 ICU/M35. Table 1 summarizes baseline characteristics. The frequency of guideline adherence for the F-NHR, F-HR, ICU/M35 groups, respectively, was 11 (39%), 4 (8%), and 4 (29%) patients. For the F-NHR group, ceftriaxone (CRO) was most prescribed (n=17, 61%). For the F-HR group, 18 (36%) received CRO; 16 (32%) received vancomycin and piperacillin-tazobactam. In the ICU/M35 group, 13 patients (93%) received meropenem plus linezolid, and 11 (79%) also received caspofungin.

No methicillin-resistant *Staphylococcus aureus* or carbapenem-resistant *Enterobacterales* were isolated. Two patients (14%) in the ICU/M35 group experienced *C. difficile* infection within 30 days of antimicrobial initiation.

**Table 1.**
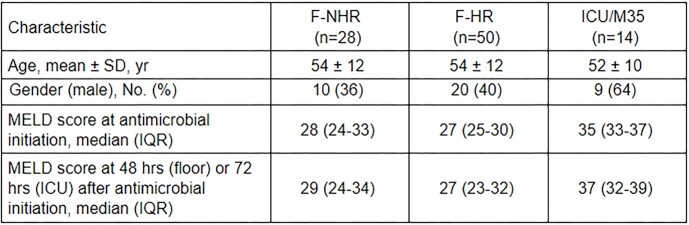
Baseline characteristics among guideline cohorts

**Conclusion:**

Our study showcases opportunities to optimize internal ESLD antimicrobial protocols and curtail unnecessary antimicrobial exposure. Study limitations include a small cohort at a single center. Future directions include data dissemination and protocol updates.

**Disclosures:**

**David Quan, PharmD**, Mallinckrodt Pharmaceuticals: Advisor/Consultant **Sarah B. Doernberg, MD, MAS**, Basilea: Clinical events committee/adjudication committee participation|F2G: Grant/Research Support|Genentech: Advisor/Consultant|Gilead: Grant/Research Support|Janssen/J+J: Advisor/Consultant|Pfizer: Grant/Research Support|Regeneron: Grant/Research Support|Shinogi: Clinical events committee/adjudication committee participation

